# Universal health coverage necessitates a system approach: an analysis of Community-based Health Planning and Services (CHPS) initiative in Ghana

**DOI:** 10.1186/s12992-018-0426-x

**Published:** 2018-11-09

**Authors:** Abraham Assan, Amirhossein Takian, Moses Aikins, Ali Akbarisari

**Affiliations:** 1 Department of Health Management and Economics, School of Public Health, Tehran University of Medical Sciences, Tehran, Iran; 2 Global Policy and Advocacy Network (GLOOPLAN), Accra, Ghana; 3 Department of Global Health and Public Policy, School of Public Health, Tehran University of Medical Sciences, Tehran, Iran; 4 Health Equity Research Centre (HERC), Tehran University of Medical Sciences (TUMS), Tehran, Iran; 5 College of Health Science, School of Public Health, University of Ghana, P. O. Box LG 13, Legon, Ghana

**Keywords:** Sustainable health development, Health System, Universal Health Coverage (UHC), Community Health, Community-Based Health Planning Services (CHPS) initiative, Ghana

## Abstract

The 9th Global conference on health promotion (Shanghai 2016) reaffirmed the role of primary health care (PHC) in achieving the 2030 Sustainable Development Agenda. Gaining much international recognition, the community-based health planning and services (CHPS) initiative is considered one of the pragmatic strategy in delivering on the promise of universal health coverage (UHC) through the PHC strategy, in Ghana. Yet, certain key factors threaten its successes – renewing the relevance of this study to present the barriers to and facilitators of the initiative. According to our findings, CHPS contribution particularly in bridging geographical access to health cannot be demeaned. Nevertheless, the full functioning of the initiative is limited by factors centered on the following themes: health governance and leadership, provision of services of quality, financial risk protection strategies targeting public health, information and care continuity, and the right mix of trained health professionals of even distribution across communities. Addressing the challenges of CHPS demand a system-approach. Substantial progress is more likely to emerge with improved leadership especially on the part of Governments to take bold political step to provide adequate financial and material resources. However, much will be achieved when stakeholders including the community work in synergies, to manage competing priorities by focusing on the core values and goals of CHPS.

## Introduction

The 2030 Sustainable Development Agenda pictures a new era for public health, including target to achieve universal health coverage (UHC) [1]. However, global reports have masked inconsistencies in progress between and within countries, suggesting much efforts to first keep effective balance between the political, economic, social landscape, in order to achieve set targets [2] – necessitating an improved national health system that promotes all-inclusiveness, transparency and participatory processes (rights-based approaches) of ensuring accountability in service delivery [3]. Hence, the international community, governments, and other concerned institutions have shown increasing support to reduce health system constraints – an approach that is anticipated to ensure a more focused policy change to facilitate achievement of UHC [4, 5].

In Ghana, health service is delivered following a three-tier system– the tertiary, secondary and the primary levels. The tertiary level is the apex of referral system while the secondary level is concerned with the provision of clinical and diagnostic care – often centered around the regional and district levels. The district level is further divided into sub-districts, which incorporate the community-level health delivery system. Public health services are delivered through a hierarchy of tertiary hospitals, regional hospitals, district hospitals, health centers and clinics, and community health posts. Health posts are the first level of primary care for rural areas, which include a Community-based Health Planning and Services (CHPS) strategy. The Ministry of Health (MOH) is charged with the responsibility of formulating policy, monitoring and evaluation, resource mobilization and regulating health service delivery in the country. The Ghana Health Service is the largest health service delivery body, and operates through the government-owned facilities [6].

Gaining much international recognition, the CHPS initiative serves as the national strategy to facilitate the achievements of UHC through the PHC approach, in Ghana [7, 8]. It emerged in 2000 to address geographical barriers to accessing improved health care, and today, remains one of governments’ flagship initiative targeting UHC which is target 3.8 of the SDG 3. Under the CHPS concept, service package which are mainly preventive, promotive and treatments of minor ailments are delivered closer to the door-step of clients, at the community level. The initiative has been able to distinctively contribute to reducing inequalities in health access, especially residents in rural and slummy areas. The major remarkable reduction in child and maternal mortality, the fairly sustained increase in antenatal care coverage (ANC) and skilled delivery, the rising performance in immunization, improvement in communities’ commitments and resource mobilization for health, are all heritage of CHPS to lead the delivery and utilization of improved health services, to reduce health inequities. Yet, the sustainability of the CHPS strategy is not certain. Significant challenges remain all too prevalent across the CHPS initiative thereby threatening the PHC of Ghana. For instance, the implementation of CHPS is not meeting the expected outcomes, partly due to inadequate transport and equipment/medicines, poor supervision of CHPS programme, weak or destroyed health infrastructure, physical inaccessibility, insufficient trained community health workforce and high attrition rate, cultural and behavioral beliefs, poor referral support, and financial insufficiency and its negative implication on the national health insurance scheme (9). Although, there is ample literature on obstacles to UHC in Ghana, it is largely unknown the extent system research is fully used to comprehensively guide local health policy action targeting UHC [5]. This study aimed to explore the barriers to and facilitators of CHPS in Ghana, through a systems-centric perspective. We envisage findings of this study can serve as evidence-based strategies to accelerate effectiveness of primary healthcare system, and to inform policy choice on Ghana’s health system, and other countries especially the low and middle-income countries (LMICs) aiming to achieve UHC.

## Methods

### Study design, sampling, and data collection

We used a qualitative design with a purposive and snowball sampling approach to interview stakeholders. There are several purposeful sampling strategies. However, for the purpose of documenting unique or diverse variations that have emerged in adapting to different conditions, and to identify important common patterns that cut across variations, we resorted to the maximum variation approach [9]. Participants, mainly, policy makers of the CHPS initiative, managers of CHPS compound and health centers, politicians, academics, health professionals, technocrats, and community health management committee members experts, were recruited across four regions of Ghana –to represent the diversity of the country in terms of socioeconomic status, cultural, religion, geographical position, and level of performance of CHPS in the country [10–12]. A total of 67 participants were interviewed – national level (5 participants), regional (11 participants), district (9 participants), and the sub-district / local levels (42 participants) – refer Table 1 for list of interviewees and their characteristics, and study settings. In-depth face-to-face interviews were conducted until saturation was reached, from April 2017 until February 2018. A pre-tested separate interview guides – designed for participants at each level were used. Each interview lasted between 25 and 45 min. Interviews were conducted in English, and tape-recorded.

**Table 1:** List of interviewees and their characteristics, and study settings

Region Levels	Central Region	Greater Accra Region	Upper East Region	Volta Region	Total
National Level	Head of CHPS (1), Head of monitoring and evaluation (1), other senior officials (2) at Policy, Planning, Monitoring and Evaluation unit at the Ministry of Health, and Renowned researcher on CHPS (1).				5
Region Levels	*Cape Coast* Deputy regional director for health administration and support services (1); Regional director for public health (1); Senior public health nurse (1); Head of service training (1); Regional health information officer (1); Regional human resource manager (1)	*Accra* Regional CHPS coordinator & deputy director of nursing services (1)	*Bolgatanga* Regional CHPS coordinator (1); Senior reproductive and Child Health (RCH) (1); Information officer (1)	*Ho* Regional CHPS coordinator (1)	11
District /Municipal Levels	*Assin North District* Municipal health director (1) Municipal CHPS coordinator (1) Municipal nutrition technical officer (1); Community mental health officer (1)	*Kpone Katamanso District* District health director (1)	*Bolgatanga Municapal & Kassena Nankana West District* Municipal CHPS coordinator (1); District CHPS coordinator (1); Nursing officer (1)	*Ho municipal* Municipal CHPS coordinator (1)	9
Sub-district/Local levels	*Assin Endwa CHPS compound* Community health nurse (CHN) (1); Head of facility (1); Enrolled nurses(EN) (1); Community health management committee (CHMC) – Traditional leader (community chief) (1), Assembly member (1); Other opinion leaders (2)	*Appolonia CHPS compound* General nurse (1); CHN (1); Midwife (1); EN (1); Nursing officer (1); CHMC – Traditional committee members (2), Assembly member (1); Other opinion leaders (3)	*Yikene & Nania CHPS compound* CHN (1); EN (2); CHO (2); CHW (3); Midwife (1); CHMC – (Traditional leader (1), Religious leader (1), Assembly member (1), Community youth volunteer (2)	*Loboli & Atikpui CHPS compound* EN (3); Head of CHPS compound (2); CHOs (2); CHW (2); CHMC -Community youth volunteer (1)	42
Total	17	13	20	12	67

### Data analysis

Data was transcribed verbatim, and coded manually. The framework approach was adopted to analyze data – following these steps: familiarization, identification of thematic framework, indexing, charting, and mapping and interpretation [13]. The World Health Organization (WHO) Health Systems Framework [14] was used to guide data analysis and interpretation. The framework was considered since it has been widely used as a benchmark in health systems research – capable of providing recognizable set of building blocks (including governance, financing, service delivery, human resources) whose functions could help improve health systems in any given country [14]. Nonetheless, we tailored the framework to incorporate Social Determinants of Health (SDH) and the Community. This modification was considered due to other contextual factors such as economic, social and cultural issues which do not form of the building block – yet could influence the performance of CHPS due to its nature – and the role SDH and the community play in achieving UHC [15, 16]. Besides, CHPS is a community-driven health system strengthening initiative to facilitate achievement of UHC in Ghana [12] – hence rendering our approach appropriate in attaining study objectives. Still, we remained open to accommodate other emerging themes since most qualitative researches are interactive processes of both deduction and induction [17].

Authors (A.A. and MA) performed the first coding – i.e. listened to the audiotapes and read the transcripts repeatedly, and got immersed with the data. Next, AT and AAS also created the inter-code connections – where data relevant to the research objectives were discussed and compared. AA and AT, again did grouped the themes and subthemes in a form of charts. Core themes which were relevant to study objectives were finally selected by the entire research team. We also enhanced the trustworthiness of the study – including the credibility, transferability, dependability, and confirmability of our findings – by employing multiple methods (face-to-face interviews, document analysis, field observation) in exploring the phenomenon (method triangulation) and theory triangulation (the use of framework that incorporates several disciplines). Further, results were returned to study participants to check for accuracy and resonance with their experiences (member checks). Again, we ensured data and method transparency, and addressed reflexivity by engaging researchers from varied background [18].

## Results

The results of the study show four main themes explaining the major health system gaps within the CHPS initiative. They include: health governance and leadership, resources, information and care continuity, and clients’ satisfaction.

### Health governance and leadership

#### Community participation

The CHPS concept has stakeholder engagements effectiveness implication. Our study revealed the importance of community participation to the extent that there are trained community volunteers (supported by health professionals), who provide direct health services within the CHPS policy framework – particularly in disease surveillance and control system. There is also an established system, i.e., the Community Health Management Committee (CHMC), who are selected and supported by communities, that serve as the governing body of CHPS at the local levels. These existing structures are put in place to ensure that the initiative yields a positive impact from the communities’ perspective. Nonetheless, there are growing challenges. While volunteerism is fundamental to the concept of CHPS, it has rarely been understood by community members. The idea has been met with strong resistance due to lack of motivation for volunteers, and activities of NGO’s by paying community volunteers to support their projects. This has contributed to a widespread concern where volunteers presently demand salaries for their services or refuse to volunteer to support CHPS.

“*community participation was stronger and there were volunteers supporting our work. Today, volunteerism is no more because of the activities of the NGO’s. We don’t pay our volunteers however are given tokens occasionally. But some of the NGO’s just move into the communities without even consulting or partnering the health directorate and began to pay volunteers to support their projects. This has negatively influenced volunteerism and activities of CHPS now. Due to their legacies, community members today either request to be paid as volunteers or refuse to volunteer to support community health programs*”. – Disease Control Officer & Assistant Chief Technical Officer, Municipal Level

Concerns from volunteers were that “*it’s very discouraging getting up in the morning for work every day and at the end of the month you end up receiving no income. I have been a community volunteer for over 15 years, yet I have not seen any significant reward for my work. I understand this is a volunteering work, but, mind you we also have families”*.

#### Political commitments

Further, the concept of making health care a shared responsibility of the governments and the community (through the CHPS initiative), in practice, hasn’t fully yielded the expected outcome – and is largely due to low political commitments to ensure the functionality of CHPS compounds, by equipping facilities with needed equipment, medicines, and technology. Instead, governments are more interested in the construction of the CHPS compounds, since it has been highly projected as a political achievement and a basis for wining vote.

*“Governments are committed in constructing structures. Political bodies enjoy the benefits of counting structures as political achievements rather than improving the functionality of the CHPS facility – i.e. by providing equipment, logistic et*c. *Hence, there are several unequipped CHPS structures”.* – Regional Director

To that extent, the siting of CHPS compound has also been one of the biggest challenge. Its location has not always been well regulated. There is often a conflict of interest, as politicians (represented by the District Assembly) may want to site CHPS compound at places to fulfil a campaign promise, while the health sector from the technical viewpoint has a CHPS rollout plan where the next priority CHPS compound should be situated. Where interests conflict, the politicians, since they have the resource at their disposal normally proceed to build the facility without further consultation from the health directorates. As a result, some of these structures have not been fully patronized by the communities following its construction – they are sometimes too close to each other or a health centre or regional hospital, or may be far away from a community. In addition, it has not only accessibility challenges but to a larger extent security implication for the health staff who reside there, or not up to the standard building plan of CHPS. Besides, provision of logistics, equipment, medicine etc. become a stumbling block because the health directorates don’t have the resources to provide them, thereby delaying the full functionality of the initiative.

### Resources

#### Human resource

According to our findings, community health professionals were not inadequate. Rather, CHPS is faced with uneven distribution of community health professionals, high attrition rate, inadequate and deplorable accommodation for staff, and inadequate auxiliary health worker – especially volunteers and security personnel.

*“one of the major challenge though some see it to be a merit is that, some of the CHPS zones are over staffed. Sometimes you get to a place and you will find over five staffs stationed there while other compounds have lesser personnel than required”. –* Head of CHPS, Regional Level.

Further, CHPS compounds lack professional security personnel. As a result, there is a challenge of health professionals accepting to stay at the remotest part of the community, or refuse to attend to client late in the nights, or few vacating their post, citing security concerns. However, some communities and often in partnership with District Assembly do recruit and pay tokens to some community members (who are not professionally trained as security persons) to function as security personnel at the CHPS compounds. Yet, such communities are few and those they recruit do not regularly go to work.

*“I rarely attend to unfamiliar voice at night, especially a male voice. I can’t differentiate a client from an armed robber. Under normal circumstance a security guard is responsible to call us to attend to clients at the dawn, should needs arise. Besides, I am the only person staying on the compound and should anything negative happen the administration will tell me I have been assigned work from morning to 4pm. I will be blamed, so why risk my life”. –* Community Health Officer

#### Financial and material resource

According to our findings, the health system of Ghana is faced with insufficient financial resources, and has contributed to governments’ inability to commit financially to the CHPS initiative. According to a senior human resource manage, “it is becoming difficult posting security personnel to the CHPS compounds. There is no financial clearance to recruit security personnel, and even for most supporting workers, it is a growing challenge even within the regional facilities and the hospitals”.

Besides, inadequacy of financial resource has contributed to lack of consistent capacity building, to the extent that, even, where training programs are organized, there are instances the full training modules are shortened – threatening the quality and sustainability of training programs. In a sample statement by senior CHPS official at the regional level, “*fourteen modules were supposed to be covered in two weeks but at a point we had to forgo the fourteen modules due to lack of funding. Because completing all the fourteen modules in three days instead of two weeks is virtually impossible*”. That is, “…the initiative is encountering high attrition rate, as against low capacity building programs. In addition, staff are not well motivated to stay on the field. So, often, they might even be trained but may leave right afterwards to further their education, to receive improved salary.” RCH Officer, Regional Level (Former Community Health Professional). Moreover, CHPS do not have the required financial assistance to acquire the needed tools, equipment, medicines, and logistics to enhance service delivery. As an example, and in a statement by a Regional Director, “there has not been any kind of major mobilization and deployment of motor bikes to CHPS zones for the past five years, of which it has negatively influenced service coverage”. The reason being that, to a meaningful extent, almost 80% of the work of the community health professionals is public health – i.e. requiring staff to move out of the static facility into the homes, communities, and outreach points, to render services. Hence, those in the remote areas are constrained due to transportation cost, and availability and ease in resorting to commercial motor bikes – because as they go out, the move at least in pairs.

Certain financial conditions have negatively affected the effectiveness of the national health insurance scheme in ensuring all clients can get access to healthcare, irrespective of their financial position. First, the scheme requires clients to be enrolled. But, still, there are several clients who are not enrolled thereby preventing them from accessing free health care. More importantly, the CHPS initiative has not been well integrated into the NHIS: First, not all communities within a CHPS zone can have a CHPS compound (a physical structure). Some must be functional (i.e. demarcating a CHPS zone by allocating a community health officer to work there, without a physical structure), and under such circumstances both clinical and public health services must be paid by the clients because there is no avenue to accredit such activities – i.e. only standing facilities which are “acting as clinic” can be accredited. Meaning, CHPS compounds need to be accredited before it can benefit from the NHIS. Implying that, until accreditation (which often it takes a longer time), clients must pay for services they receive – both clinical and public health services. Besides, even after accreditation, it is only the clinical serves that is covered by the insurance scheme. Further, it takes a longer period for the insurance authority to reimburse facilities their insurance claims – sometimes beyond 6 months – which possess a lot of financial challenge in the management of health facilities. Hence, health care providers end up charging clients’ fees for some services although per policy it is supposed to be free. So, basically, although CHPS is public health focused, there is limited efforts in pushing forward the public health agenda within the CHPS framework, to ensure that clients are not denied of service due to financial reasons.

*“the insurance authority is not reimbursing health facilities their health claims as required. Not at all! Forget about the political noise. Again, some of the CHPS zones are not accredited hence clients need to pay for the services they receive. And, even with the accredited facilities, most public health services which is a major component of CHPS service package are not being covered by the scheme”.* Director for Public Health, Regional Level.

Besides, participants repeatedly reported lack of medicines at the regional medical stores, or the inability of health facilities to procure the needed medicines for their facility from the regional medical stores (even if they are available at the store), due to inability of the National Health Insurance Authority to reimburse health facilities their insurance claims on time, to pay outstanding debt. In view of that, medicines are prescribed for the clients to be purchased from private pharmacy shops on regular basis.

*“During the early years of CHPS the health insurance was very effective – you go to any health facility and all your health costs are virtually being covered by the NHIS. That was very helpful because the community members don’t have the money and for that matter all were willing to enroll under the insurance scheme. But nowadays, Health Insurance Authority is unable to reimburse health facilities on time. As a result, managements of health facilities find it difficult raising money to sustain the facility. Also, currently it’s becoming difficult to convince someone to enroll or renew his or her health insurance scheme. Clients keep telling us that whatever the situation they must pay for the medicines and therefore there is no need to acquire health insurance. In fact, they are always complaining but there is nothing we can do at our level.” – Head of CHPS, Regional Level*

### Information and care continuity

#### Referral system

Community health professionals are mandated to refer complicated cases, or services beyond their scope of service to the higher level of care. Nonetheless, certain key factors were found threating the effectiveness of the referral system. First, it takes a longer time for clients to adhere to referrals. That is, community member rarely accepts to receive care at higher levels. Reasons given include poor reception by health professionals and long waiting time at such levels. As a result, it requires extra effort for health professionals at the community level to convince clients to continue treatment at the next level of care. Again, ambulance is one of the means of transport to ensure that referral system works effectively. Yet, the study revealed lack of effective ambulance system to transport clients from the community level to the other levels of care. Problems are that, they are often not accessible. Hence, clients, as much as possible, through the cooperation with health professionals, try to use available commercial transport within the community. The challenge in resorting to commercial transport is that, clients must fund their transport which often they lack the money to do so – posing extra indirect cost of receiving care. Besides, resorting to commercial vehicles is also challenging and associated with major uncertainties which delay the entire referral processes. Again, the use of mobile phones was found relevant during referral processes and follow-up. Nonetheless, this is limited by poor network connection.

“......*when my client is referred to another level for treatment, that ends there – I am less aware whether he/she arrived or not, is responding to treatment or not, discharged or did not survive. It is not that I care less – in fact, I care to know but that system isn’t effective to support us to that extent*”. – Community Nurse.

Extra findings from the health professionals in that, members of the community also recognize CHPS compounds as their own property and for that matter feel much comfortable seeking for health care at the CHPS compound compared to the bigger hospitals. As a result, many try to even resist when transferred to the bigger facility. According to a senior community health professional with over 10 years working experience at the community level, “*from critical observation clients that regularly visit the CHPS compounds rarely would like to go to the bigger hospitals even when referred – not that they don’t want to receive assistance from the bigger facilities – they see CHPS compound as theirs and for that matter feel much comfortable seeking for health care there compared to the bigger hospitals.”*

### Clients’ satisfaction

Clients reported of positive attitude of community health professionals, and improved satisfaction of services they deliver. They testified of enough trust and confidence in sharing sensitive information with the community health professional, especially during home visit. According to the community members, “*an ordinary home visit by a health professional make me feel like almost half of my problems solved. It is just like being a church member or a student and suddenly your pastor or teacher visits you at home just to inquire of your health or wellbeing. The feeling is awesome, just like being treated as a king. The same applies to when community health professionals visit my home to talk to my household about health. You feel being cared for – a feeling of a unified bond with health professionals which we cannot get anywhere else, not even the bigger hospitals – the long queue and the attitude of most health professionals alone there is very appalling.”* – Community member. Further reasons given by clients were that, they always meet different health professionals anytime they visit the bigger hospital – people they are not even familiar with, hence makes it difficult for them to share certain information with them. On the other hand, they are much acquainted with the community health personnel – they often meet them either at the CHPS compounds or their homes. In view of that, it is of ease to them to disclose sensitive information to community health professionals. Besides, according to the community members, the health professionals seem to know every detail of them since they live within their community and therefore would even be unwise in any attempt to uncover any information pertaining to their health.

Although clients showed enhanced satisfaction with the services provided by community health professionals, from the technical perspective, certain key factors do compromise the quality of care or services provided. According to health providers, quality of services provided used to be better, but that isn’t the case now due to lack of equipment, and needed technology to aid improve the quality of services provided at the CHPS zones. Again, to the senior officials, there are several problems in terms of the capacity of staff deployed to CHPS zones. That is, most of the health professionals at the community level do lack the community mobilization capacity, resource mobilization capacity, and community entry skills, to effectively render quality service.

“*technically, not all of community health professionals have the capacity to manage community issues – particularly community mobilization capacity, resource mobilization capacity, and community entry skills. Besides, CHPS compounds lack basic equipment and the logistic support to reach out to their clients. Hence, not adequately prepared to provide services at the CHPS zones. Based on these, overall, I can say that quality of care is an issue. That is, the condition under which the health professionals work you cannot expect a 100 percent outcome– it is not too bad neither can it be 100 percent good”.* – Senior policy maker, national level

## Discussions

Fundamental to achieving UHC is a sustainable health system [19]. However, due the existing disparities between and within countries, attaining UHC will necessitate all-inclusiveness, a participatory processes and interactions between health systems and the population [3]. Besides, there is increasing evidence where most successful systems use population-driven interventions to reshape PHC to address gaps pertaining to availability, accessibility, and affordability of high-quality health service – renewing the importance of CHPS in ensuring healthy lives and well-being of the people of Ghana [11]. Meaning that, the rudiments and prospects for health cannot be left in the hands of governments alone. It demands robust joint action by all stakeholders, including the governments and the community, and other institutions even beyond the health sector. CHPS dwells on a shared responsibility concept (on the part of the governments and the communities), particularly in health service delivery, and resource mobilization and management. Although, decisions are organized around the needs, expectations and the perspectives of a defined community, basically, governments have decisive responsibility for ensuring the overall performance of health systems [20]. Yet, based on findings of the study, governments efforts toward provision of basic equipment, medicines, technology and required logistics in sustaining the initiative is minimal. Part of the reason is that, political parties have conflicting perspectives on addressing health needs based on the core values and goal of CHPS, and achieving their institutional mandates. In view of that, governments major role is shifting towards construction of CHPS structures over improving the functionality of the CHPS facility by providing equipment, logistic. The reason being that, the existence of a physical structure (CHPS compound) serve a credible basis for voting politicians into power at the community level. Political actions – i.e. improved governance for health (at all levels) is crucial for better health care – especially in resources allocation and services delivery. However, in circumstances like this, directly engaging the society can contribute to instilling values that strengthen accountability [21]. Yet, according to our findings, although CHPS is community-driven, the system lacks effective structures to assist communities to hold Governments fully answerable to their responsibilities. In our perspective, emphasis is rather placed on community’s commitments to their health developments – people who lack the needed resource, and mobilization skills to effect positive change.

Effective health system requires a mix of human and material resource [22]. Even though CHPS lack needed medicines and equipment, human resource (community health professionals) are not scarce. Instead, larger proportion of them have not been adequately trained – they lack community mobilization skills, resource mobilization capacity, and community entry skills, to effectively render improved service. Also, community health professions are unevenly distributed – either too many or few for a geographical area. Further, insufficient auxiliary workers (especially community volunteers and security personnel) have slowed down the work of health professionals. However, improving the capacity of health workforce, and provision of material resources in its appropriateness, require financial commitments. That is, variety of resource – including – appropriate trained personnel, sufficient quantity of suitable facilities or condition in which to provide service, adequate financing for primary health care services, accessibility of services to the population, adequate information systems and effective governance mechanisms, are all pre-requisite for a health systems to achieve its goal [23]. In view of that, although community members showed improved satisfaction of services provided by community health professionals, especially in ensuring clients confidentiality, and addressing waiting time, from the technical perspective, quality of services provided could be compromised due lack of financial resource to provide the needed equipment and technology, and to train health professionals, to provide quality services.

Generally, financial resources for health care are finite – suggesting health systems to be strategic to achieve effectiveness by preventing inappropriate resource allocation [20, 24]. Studies have revealed where in some countries a single tertiary care hospital absorbed almost 20% or more of the overall budget assigned to the Ministry of Health – inappropriately serving the more affluent urban sector at the cost of failing to deliver a more effective clinical care through health facilities decentralized at urban and rural communities [25]. Failure to allocate resource properly to fully address inequalities tends to be one of the most pressing problem preventing both the High and LMIC from achieving desired outcomes – even where there are high financial commitments to health systems – because often public funds are expended on low-cost effectiveness interventions over a more cost-effective ones, or is exhausted in training too many specialist, whose services are not the pressing needs of the society or doctors who are less likely to practice at places they are needed most, predominantly in rural communities or among the poor [26]. These signify that, where there is insufficiency or uneven distribution of national resource and program implementation, there is a greater possibility for the poor to receive lower quality of care. Hence, with any system, effective healthcare based on the recommendation of the WHO should ensure that: most relevant health concerns of greatest needs are prioritized; there is sustainable funding to provide improved equipment, facilities and trained health care providers, to ensure consistent delivery of high quality service [20]. Yet, while each of the value of quality, equity, and cost-effectiveness is important, efforts in producing any of these values independently may affect the other positively or negatively. As a result, objectives of health systems should target achieving equilibrium (harmonious integration and balance of these values) to maximize impact [26, 27].

Although it tends to be challenging, it is achievable delivering high quality and cost effective essential clinical and public health services to the entire population – through a more comprehensive system of health service delivery, where a balance of needs of all forms (promotion, prevention, treatment, rehabilitation and palliation), are provided at the least cost, at any place (rural or urban), and time, without financial insolvency [23]. Besides, it has been established that vertically organized services are not efficient, and therefore, addressing present public health challenges require more integration [28]. Further, based on the WHO World Health Report 2010, frequent segregation and lack of coordination of public preventive services and individual medical care creates additional inefficiencies – specifying how it contributed to 20–40% of total heath expenditure [24]. Yet, according to our study, public health services which is a major component of CHPS service package are not well integrated into the NHIS of Ghana – posing further financial barrier to health care. Also, although CHPS service package is mainly prevention, promotion, and treatment of minor ailment, the initiative lacks needed ambulatory services to facilitate referral processes in contributing to comprehensive delivery of services. Again, challenges related to availability and reliable communication technology has altered the way health professionals at the community level interact with their clients, and especially with their fellow professionals at the higher referral levels – posing threats to information and care continuity, and quality of care [29].

### Strengths and limitations

This study has three major strengths:

1. Due to the complexities and cost involved in health system research, few of such have been conducted to influence local policy actions. This study is the first of it kind to holistically explore the CHPS initiative following the launch of the SDGs.
2. The stratified data collection strategy, the diversity of study settings and participants, helped provide broader understanding to the study.
3. The model used – the WHO health System Framework is also regarded the widely used and most comprehensive health system tool capable of accommodating wide range of perspectives, including social, political, economic, and phycological, for practical interpretation to health system development.
4. We also enhanced the trustworthiness of the study – including the credibility, transferability, dependability, and confirmability of our findings – through member checks, method and theory triangulation, data and method transparency, and addressed reflexivity through thorough engagement of researchers from varied background.
5. However, there remain a key gap. The snowball sampling approach may have led to loss in diversity of experiences due to the personal referral system.

### Implications for policy and research

- CHPS contribution particularly in bridging geographical access to health cannot belittled. Nonetheless, for Ghana to move more quickly towards UHC, improving on other components of UHC including provision of quality of services is critical. Also, CHPS should be well integrated into the NHIS – to ensure that public health services (which is the major activities of CHPS) are covered by the insurance schemes while accreditation processes of facilities and reimbursement of health facilities their health claims do not also pose extra indirect financial barrier to clients when accessing health.
- Productivity of CHPS is also threatened by inadequate funds to train health workers and other actors especially at the community level. An Open Online Educational Resource for community health professionals could serve as a platform for efficient and consistent capacity building, especially for community health professionals and other supporting staff.
- The initiative’s ability in promoting accountability and commitments of community members towards their health development has yielded meaningful outcome, but could be maximized if mechanisms are in place to empower communities to also ensure that governments remain committed to providing health needs of its citizens to the fullest.
- An effective communication technology strategy and provision of ambulance have the potential to also improve care and information continuity at the PHC level.
- To maximize impact, objectives of health systems should target achieving equilibrium, i.e., harmonious integration and balance in quality, equity, and cost effectiveness.
- The NHIA should develop guidelines to accredit CHPS entities that do not have a physical facility to help better fund services in under-resourced areas.
- Since data was gathered from interviewees at different levels and places, and perchance with diverse socio-political ideologies and background, we cannot be certain of participants’ primary rationale of responses specified. Hence, findings should be interpreted with carefulness.
- CHPS is a sub-health system of Ghana. Further research is needed to assess the challenges of the other sub-systems especially in ensuring information and care continuity across levels, and in ways they inform each other.

## Conclusion

The 9th Global conference on health promotion (Shanghai 2016) undoubtedly reaffirm the role of PHC in achieving the 2030 sustainable development agenda. According the findings of the study, CHPS is still of particular importance to a well-functioning PHC – in delivering on the promise of UHC and sustainable health development in Ghana. However, CHPS is faced with several health system gaps, centered on: health governance and leadership, resources, information and care continuity, quality of care, and financial barriers to health. The initiative can obtain more value for its investments to best address the health challenge of the people of Ghana, through a systems-centric approach. Substantial progress is more likely to materialize through improved leadership especially on the part of the governments in taking bold political action to provide financial and material resources – but much will be achieved when stakeholders work in synergies to manage competing priorities while focusing on core values and goals CHPS seeks to achieve.
